# Photodynamic therapy in a pleural cavity using monte carlo simulations with 2D/3D Graphical Visualization

**DOI:** 10.17352/2581-5407.000045

**Published:** 2022-09-29

**Authors:** K Beeson, E Parilov, Mary Potasek, T Zhu, H Sun, D Sourvanos

**Affiliations:** 1 Simphotek, Inc, 211 Warren St, Newark, NJ 07103, USA; 2 Perlman School of Medicine, University of Pennsylvania, Philadelphia, Pennsylvania, USA; 3 Department of Periodontics, School of Dental Medicine, University of Pennsylvania, Philadelphia, Pennsylvania, USA

Cancer therapy using Photodynamic Therapy (PDT) has been investigated for some time [1,2] and now it is a growing area of interest in clinical trials [3]. Monte Carlo (MC) simulations were used for early laboratory studies [4,5] for analysis in PDT. Various improvements in the MC method have advanced the field in recent years. For example, Yassine, et. al. [6] have optimized PDT with custom cylindrical diffusers; Cassidy, et.al. [7] have developed a robust MC method; whereas, Fang and Yan [8] and Young-Schultz et. al. [9] ported MC to Compute Unifi ed Device Architecture (**CUDA**) that run on Graphics Processing Units (**GPU**). To date, there is a lack of very fast (a few minutes or less) computational methods for treatment planning in the clinic. Simphotek (**Stk**) [10–12] and other references in [13], including references therein, have developed various MC-based methods that can simulate the light fluence in PDT in near real-time.

The Perlman School of Medicine (**PSM**) has investigated PDT in the pleural lung cavity of several patients in a Photofrin–mediated study [14] and developed an IR navigation system for clinical use [15,16]. The analysis of the PDT dose data for 19 patients has been published recently [3]. However, due to the large surface area of the pleural lung cavity, a series of multiple stationary light sources is needed. **PSM** is currently developing an 8-detector system for treatment in the pleural cavity. While multiple fixed detectors can be used for dosimetry at a few locations, an accurate simulation of light fluence and fluence rate is still needed over the entire cavity. This makes it difficult for treating physicians to visualize the multiple light fluence/fluence rate simulations. As a the result, **Stk** extended its GPU-based MC simulation tool, as a part of Dosie^™^ simulation software, for modeling the light transport in intracavity PDT (**icav-PDT**) to include a dose-cavity visualization that allows a user to inspect the dose maps in real-time over the treated cavity in 3D.

As being a part of an emerging PDT Explicit Dosimetry System (**PEDSy**), the performance of this new Stk’s CUDA-based implementation, called PEDSy-MC, has been demonstrated on a life-size lung-shaped custom-printed phantom for testing the icav-PDT navigation system at the **PSM** [15,16]. Fluence calculations completed in under a minute (for some cases) or within minutes have been achieved [17]. In addition, results within a 5% error of the analytic solution for multiple detectors in the phantom were accomplished. Research supported by [18].
